# Development and Characterization of Methylene Blue Oleate Salt-Loaded Polymeric Nanoparticles and their Potential Application as a Treatment for Glioblastoma

**DOI:** 10.4172/2157-7439.1000449

**Published:** 2017-07-31

**Authors:** JM Castañeda-Gill, AP Ranjan, JK Vishwanatha

**Affiliations:** 1 Harold C. Simmons Comprehensive Cancer Center, University of Texas Southwestern Medical Center, Dallas, TX 75390, USA; 2 Institute for Molecular Medicine and Institute for Cancer Research, University of North Texas Health Science Center, Fort Worth, TX 76107, USA

**Keywords:** Methylene blue, Nanomedicine, Glioblastoma, Brain tumor, Neurotherapeutic

## Abstract

Glioblastoma (GBM) is an aggressive, grade IV brain tumor that develops from astrocytes located within the cerebrum, resulting in poor prognosis and survival rates following an accepted treatment regimen of surgery, radiation, and temozolomide. Thus, development of new therapeutics is necessary. During the last two decades, methylene blue (MB) has received increased attention as a potential neurotherapeutic due to its duality in brain cancers and neurodegenerative diseases. While MB is capable of easily permeating the blood-brain barrier, its therapeutic concentrations in GBM are known to induce off-target cytotoxicity and thus, another mode of drug delivery must be considered. To this end, encapsulation of formerly unusable compounds into nanoparticles (NPs) made from the biodegradable/biocompatible, FDA approved co-polymer poly (lactide-co-glycolide) (PLGA) has been more commonplace when developing novel therapeutics. In this study, we formulated and characterized Pluronic F68-coated PLGA NPs containing a sodium oleate conjugate of MB (MBOS) via solvent displacement. Conjugation of sodium oleate to MB was shown to reduce its release from PLGA NPs compared to unmodified MB, leading to potential improvements in drug accumulation and therapeutic effectiveness. Our drug-loaded NP preparations, which were ~170 nm in size and had drug loading values of ~2%, were shown to reduce cell viability and cell compartment-specific, as well as overall cell, functions equivalenty, if not more so, when compared to free drug in two GBM cell lines. Following bio-distribution analysis of free MBOS compared to its nano-encapsulated counterpart, drug-loaded NPs were shown to more effectively permeate the BBB, which could lead to improvements in therapeutic effectiveness upon further examination in a tumor-bearing mouse model. Based on these results, we believe that the further development and eventual utilization of this nanoformulation could lead to an effective GBM therapy that could extend patient survival rates.

## Introduction

According to statistics from the American Cancer Society (ACS), of the estimated 1.7 million newly diagnosed cancer cases within the United States this year, nearly 1.4% (23,130) will be due to tumors of the brain and nervous system; additionally, of the 580,000 total cancer deaths, approximately 2.4% will be attributed to the aforementioned anatomical areas [1]. Although these figures are significantly lower than those of more recognized cancer types (breast, colorectal, lung, pancreatic, and prostate), brain-associated cancer prevalence is rising due to increased resistance to conventional treatment methods - a combination of surgery, chemotherapy, and radiation - leading to significantly lower survival rates [2–4]. Glioblastoma (GBM), a rapidly developing grade IV brain cancer that originates from supportive glial cells called astrocytes, resulting in its classification as an astrocytoma, is located primarily within the cerebrum and considered the most common and aggressive primary human brain tumor due to poor prognosis (15 months maximum post-diagnosis with treatment) and substandard five-year survival rates (less than 5%) [2,3,5–10]. While GBM can affect individuals of all ages, it occurs more frequently in adults, primarily middle-aged people between 45 and 65, with a gender disparity favoring men, as well as a slight racial discrepancy towards Caucasians [2,3,11–15].

GBM, like other cancers and cells undergoing rapid proliferation, depend on an inefficient energy producing process called aerobic glycolysis to generate adenosine 5’-triphosphate (ATP) and other metabolic precursors for successive colony expansion through a phenomenon referred to as the Warburg effect [16–19]. In the Warburg effect, highly proliferative cells, including cancers such as GBM, exhibit irregular mitochondrial behavior that results from disjointed energy metabolism, leading to a dependence on cytosolic glycolysis for energy [20–23]. While decades of research have established the Warburg phenomenon and associated mitochondrial dysfunction as a consequence of compounding genetic mutations, the concept continues to be an intriguing aspect in the development and progression of cancer, specifically GBM, and thus, provides an avenue for the development of novel therapies.

Methylene blue (MB), also referred to as methylthioninium chloride, is a water soluble compound that was discovered in the late 1870s and originally used as a histological dye. MB, which has a proven safety record and demonstrated versatility in clinical applications, has been used to treat maladies ranging from chemotherapy-induced encephalopathy and can act as a photodynamic therapy (PDT) in cancer patients to more historical conditions such as cyanide poisoning and malaria [24–31]. In the case of various diseases, including GBM, MB has been shown to target dysfunctional mitochondria by acting as an electron carrier via its reversible photoreduction to inactive leucomethylene blue (LMB) to aid cytochrome c reduction, bypass complex II, increase oxygen consumption, and increase the production of reactive oxygen species (ROS) [32–41]. While MB shows promise as a neurotherapeutic due to these benefits, as well as its ability to easily permeate the blood-brain barrier (BBB), the challenge arises when administering MB to patients. In order to achieve the necessary accumulation of drug concentration for treatment, a higher-than-required dose must be given, leading to potential overmedicating and off-target toxicity [29]. As a result, additional drug delivery methods must be considered.

Traditional modes of drug delivery across the BBB involve disruption of the BBB, which if still intact during the disease state, can lead to infection; lipidation of small molecules; and delivery of anti-sense or non-viral DNA [42]. Due to recent advances in nanotechnology, its applications have been considered as possible mechanisms for more effective drug delivery across the BBB, ranging from encapsulation in liposomes and polymeric nanoparticles to direct conjugation to antibodies [43–49]. Over the past few decades, nanoparticle drug development has grown because of its wide versatility of applications and formulations. In accordance with the previous statement, nanoparticle delivery of drugs for GBM has grown due to the need for more effective treatments that can maneuver the BBB. While numerous options are currently available for developing nanodrug delivery systems, including liposomes; solid lipid nanoparticles; polymeric nanoparticles; hybrid nanoparticles; dendrimers; and nanotubes, this study focuses on the application of polymeric nanoparticles, derived from the synthetic co-polymer poly (lactide-co-glycide) (PLGA). PLGA was the first FDA-approved co-polymer for medical use, is biocompatible/biodegradable via its non-enzymatic hydrolysis at its ester linkages to lactic and glycolic acids, and has confirmed brain accumulation in in vivo studies [50–53]. Some of the advantages afforded by PLGA NPs as drug delivery systems include: their ability to encapsulate numerous compounds; provide targeted drug delivery using surface functionalization with antibodies/peptides; allow tunable sizing; can be prepared from various matrices; improve therapeutic efficacy of drugs due to reduced clearance; can be used for various administration routes, reduce toxic side effects; and traverse biological barriers, including the blood-brain barrier (BBB), skin, and tight junctions of various epithelial layers [43]. PLGA NPs are thought to obtain passage through the BBB via receptor-mediated endocytosis in brain capillary endothelial cells, which results from either covalent attachment of targeting ligands or coating with certain chemicals that enable adsorption of specific plasma proteins for improved circulation and distribution [46]. Methods that are being considered in order to improve PLGA NP passage through the BBB, as well as improve tumor uptake, include active targeting via surface conjugation with antibodies or BBB receptor ligands and use of surfactants [54].

While MB-loaded NP formulations have been developed in the last several years to treat various conditions, the concern with utilizing MB in a PLGA NP is due to its high water solubility. PLGA NPs tend to encapsulate hydrophobic and lipophilic drugs more effectively than hydrophilic drugs; therefore, modifications to MB are necessary that enable prolonged entrapment within the NP until delivery to the target site without affecting its normal chemical functions. To this end, we formulated a methylene blue oleate salt (MBOS) conjugate and encapsulated it within PLGA NPs. In this study, we tested the hypothesis that encapsulation of MBOS in PLGA NPs would elicit minimally equivalent *in vitro* effects in multiple GBM cell lines when compared to free MB, as well as free MBOS, based on the reversal of mitochondrial dysfunction and supposed reduction in off-target side effects. Additionally, we sought to determine the biodistribution of MBOSNPs compared to free MB and MBOS, as a means to confirm any potential clinical applications toward improving disease progression and reducing drug-associated toxicity.

## Experimental Procedures

### Cell culture and other reagents

U87 MG (U87) was gifts from Dr. ShaoHua Yang (University of North Texas Health Science Center), while T98G cells were obtained from American Type Culture Collection (ATCC). Both were cultured as previously described [55]. MB was purchased from Calbiochem. Sodium oleate, Pluronic F68, glucose oxidase/peroxidase solution, O-dianisidine dihydrochloride, and D-(+)-glucose were obtained from Sigma-Aldrich. PLGA 50:50 DLG 8E was purchased from Lakeshore Biomaterials. Pierce protein reagent was obtained from ThermoScientific, D-luciferin sodium salt from Regis Technologies, and QuantiLum recombinant luciferase from Promega.

### Preparation of methylene blue oleate salt

In pre-weighed 250 mL glass beaker containing a stir bar, 100 mg sodium oleate (SO) was dissolved in 100 mL distilled, deionized (DDI) H_2_O. 50 mg MB was added to 5 mL dehydrated ethanol and mixed, then combined with SO solution and allowed to stir overnight at room temperature in a fume hood to prepare the MBOS solution. The following day, 100 mL chloroform was added to the MBOS solution, stirred for 5 minutes, and then placed at room temperature in a fume hood for at least 24 hours to obtain complete partitioning of the organic and aqueous layers. After achieving layer separation, the H_2_O layer containing free MB was removed, and the beaker containing the MBOS chloroform solution returned to stirrer under vacuum in fume hood for 72 hours until chloroform has completely evaporated and layer of MBOS coating remained. To determine the final amount of mg of MBOS obtained, the beaker was removed from stirring and re-weighed.

### Preparation and characterization of MBOSNPS

#### Preparation of MBOSNPs

**Figure 1:**
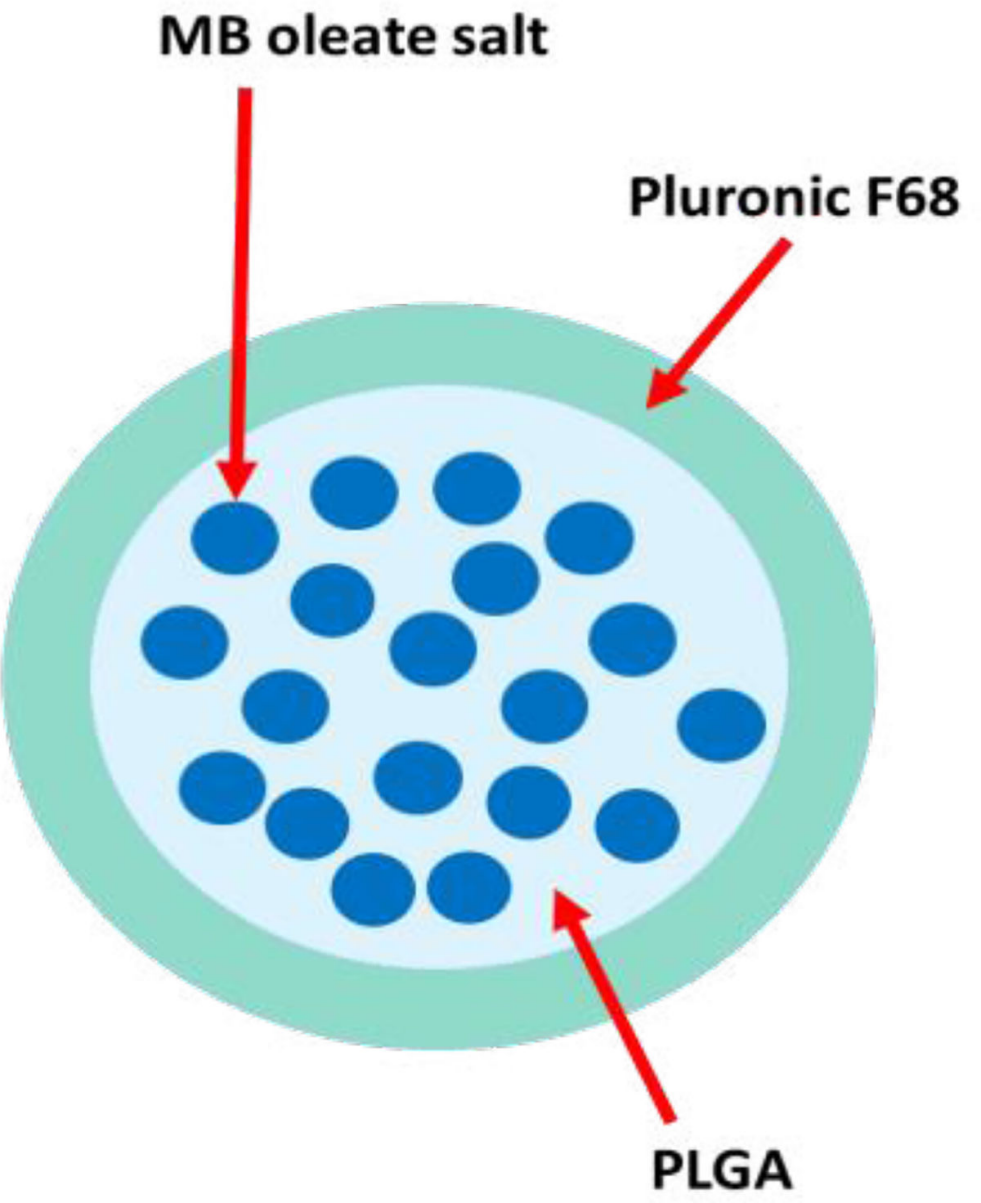
Schematic representation of MBOSNPs. MBOS, produced by chloroform extraction to reduce water solubility and release, was encapsulated within the co-polymer PLGA via solvent displacement, and subsequently coated with the surfactant Pluronic F68 and lyophilized. Abbreviations: MBOSNPs, methylene blue oleate salt-loaded nanoparticles; MBOS, methylene blue oleate salt; PLGA, poly (D,L-lactide-co-glycolide).

To generate MBOSNPs (Figure 1), 3 mg MBOS was combined in 1 mL acetone, placed on a mini vortexer until completely dissolved, then 10 mg PLGA added and returned to vortexer until PLGA was in solution. While MB and PLGA solution was vortexing, 1% PF68 in DDI H_2_O was prepared and 3 mL filtered through a 0.45 micron syringe filter. Once MB and PLGA solution was obtained, it was added to PF68 solution and placed under compressed nitrogen gas until acetone has evaporated and organic solvent odor no longer remained (~1 hour). Next, the NP sample was transferred to a 50 mL 10K cut-off Amicon tube, where ~10 mL DDI H_2_O was added, then centrifuged at 3500 rpm for 20 minutes at 4°C. Once the flow through was discarded, centrifuge/wash step was repeated 2× more for 20 minutes, then 30 minutes. Once washes were concluded, 5% sucrose in DDI H_2_O was added to NP sample to obtain 1 mL total volume. The liquid MBOSNPs were transferred to a pre-weighed cryovial, placed at −80°C overnight, and then moved to a cooled, pressurized lyophilizer (ATR, Inc.) for 72 hours. Upon removal from lyophilizer, sample was stored at −20°C until needed. Empty, blank NPs were also generated, analyzed, and tested in subsequent *in vitro* assays for comparison at the highest “treatment” concentration for drug-loaded NPs.

#### Physicochemical characterization of MBOSNPs

Prior to freezing and lyophilizing, as well as post, particle size and zeta potential were analyzed via a Malvern Zetasizer Nano ZS. Briefly, a small amount of lyophilized NPs was re-suspended in 1 mL DDI H_2_O, vortexed for 30 seconds, then transferred to a disposable cuvette (Sarstedt) for analysis of particle size (in nm) by dynamic light scattering. The sample was then transferred to a disposable capillary cell (Malvern) for analysis of zeta potential. For each NP batch, the mean diameter ± S.D. for three measurements was determined. The polydispersity index (PDI) was also quantified to establish particle size distribution.

#### Drug loading and encapsulation efficiency determination by UV/V is spectrophotometry

Drug loading and encapsulation efficiencies were determined post-production by mixing 5 mg lyophilized MBOSNPs in 1mL acetone, placing in a 37°C incubator shaker for 4 hours, then centrifuging at 14,000 rpm at room temperature for 5 minutes to precipitate residual PLGA. The acetone component containing MBOS was then analyzed via the UV/Vis spectrophotometry component of a Nanodrop (ThermoScientific) at 645 nm, with values compared to an MBOS standard curve.

#### Transmission electron microscopy (TEM)

Following acquisition of the lyophilized final product, MBOSNPs were also analyzed for shape and surface morphology via TEM. Briefly, a small amount of NP was resuspended in 1 mL DDI H_2_O, then diluted 1:10. A drop (~5 µL) of diluted, resuspended MBOSNP sample was deposited on a discharged carbon grid, allowed to dry for 1 minute, carefully blotted, and a drop of 2% uranyl acetate added with a 1-minute dry time and subsequent blotting. Once negatively stained, the NP sample was on examined under a Tecani Spirit Biotwin at the University of Texas Southwestern Medical Center’s Electron Microscopy Core Facility.

#### MB(OS) release kinetics

To determine the rate of MBOS release from the NPs, MBOSNPs containing at least 0.5 mg MBOS (~30 mg) was weighed out and added to 2 mL 1X PBS in 8 MWCO dialysis tubing. The dialysis tubing containing sample was then placed in a 250 mL glass beaker containing 100 mL 1X PBS as a “sink” under constant stirring at room temperature. At designated time points, 1 mL of 1X PBS from the “sink” was collected, replaced with fresh PBS, and analyzed at 645 nm via the UV/Vis spectrophotometry component of a Nanodrop (ThermoScientific), with values compared to an MBOS standard curve.

### *In vitro* analyses

#### Cellular bioenergetics analysis

Assay was performed according to manufacturer’s directions and as previously described [55], where U87 and T98G cells were plated at 30,000 cells/well in an XF24 plate and allowed to attach overnight. Media was replaced an hour before onset of the assay with XF24 media, and then, rotenone/antimycin A mix, FCCP, and oligomycin were diluted in XF24 media and loaded into the provided cartridge to obtain final concentrations of 100 nM, 300 nM, and 1 µg/mL, respectively. MB, MBOS, or MBOSNP treatments, at pre-determined concentration, were also inserted into the cartridge. Addition of the compounds into the medium took place at designated time points, and oxygen consumption was examined using a Seahorse Bioscience XF24 Extracellular Flux Analyzer.

#### Liquid colony formation assay

Cells were seeded into 6-well culture plates (Falcon) at a concentration of 50 cells/well in 1 mL DMEM high glucose with pyruvate (Gibco), 10% FBS, and 1% Pen/Strep. Treatments were added to each well to obtain a pre-determined final concentration in 2 mL/well total volume. Plates were incubated for 4 weeks undisturbed, and only carefully received a media change at week 3 with or without drug treatments if colonies were not visible. Following completion of the 4-week incubation period, colonies were stained as previously described [55]. Culture plates were placed on ice and gently washed twice with ice-cold PBS; next, colonies were fixed with ice-cold methanol for 10 minutes, which was removed to allow for staining; plates were then relocated to the bench-top, where the colonies were stained with 0.5% crystal violet in 25% methanol for 10 minutes, which was removed; finally, plates were washed by immersion in cold, running tap water until the water ran clear and placed upside down on absorbent paper to allow overnight drying. Stained colonies were counted, with the number and size documented.

#### ATP quantification

U87 and T98G cells were seeded into 6-well plates at 200,000 cells/well in 1 mL DMEM high glucose with pyruvate (10% FBS and 1% Pen/Strep) and allowed to grow overnight. The next day, media was replaced with MB, MBOS, or MBOSNPs at desired concentrations and analyzed at specified 24 hour increments. Following a modified protocol outlined in an ATP kit obtained from Life Technologies, cells were washed twice with PBS, then lysed with 150 uL of ATP assay buffer (500 mM Tricine buffer, pH 7.8, 100 mM MgSO_4_, 2 mM EDTA, and 2 mM sodium azide) containing 1% Triton X-100, dislodged by scraping with a pipette tip, and transferred to 1.5 mL Eppendorf tubes on ice for 5 minutes. 10 uL of cell lysate was then added in triplicate to a white 96-well plate, also containing ATP standards. Before reading the plate, 100 uL of ATP assay buffer containing 90 µg/mL D-luciferin, 20 µM DTT, and 25 µg/mL Luciferase) was added to each well. Luminescence was calculated using a Tecan Infinite F200 plate reader. Protein concentration was also determined using the Pierce 660 nm Protein Assay (660 nm absorbance), with ATP production standardized to protein values.

#### Glucose quantification

Glucose was measured per instructions from a kit manufactured by Sigma-Aldrich. Briefly, 200,000 U87 and T98G cells were seeded into a 6-well plate and incubated overnight. On the subsequent day, media was removed and exchanged with 2 mL fresh DMEM high glucose (4.5 g/L glucose) contained pre-determined MB, MBOS, or MBOSNP concentrations. After designated times, medium was removed, diluted 1:100 in glucose assay buffer (glucose oxidase, horseradish peroxidase, and O-dianisidine), then added to a 96-well plate at a 1:3 dilution in glucose assay buffer. The plate was then incubated at 37°C and 5% CO_2_ for 30 minutes, and the reactions halted by the addition of 66 uL of 12 N sulfuric acid. Absorbance values were evaluated at 540 nm on a Biotek Synergy 2 plate reader.

#### Cell viability

U87 and T98G cells were seeded at 750 cells/well in 100 µL DMEM high glucose with pyruvate (10% FBS and 1% Pen/Strep) in a black, 96-well flat-bottomed plate, and allowed to attached overnight. The following day, 50 µL media containing varying concentrations of MB, MBOS, or MBOSNP was added designated wells and incubated at 37°C overnight. The next morning, the plates were washed with 200 µL/well 1X PBS, which was removed and replaced with 95 µL calcein AM reagent at 1:1000 dilution in 1X PBS. Plates were incubated at 37°C for 5–10 minutes, protected from light, and read via a Tecan Infinite F200 plate reader at 485/530 excitation/emission fluorescence.

### *In vivo* bio-distribution

All animal studies were performed at the University of Texas Southwestern Medical Center’s Preclinical Pharmacology Core, with practices following Institutional Animal Care and Use Committee and National Institute of Health guidelines. Female mice (CD-1 background, 5–6 weeks old) were used for all bio-distribution studies. Mice were administered a single intravenous (IV) dose of either MB (in 95% PBS/5% DI H_2_O), MBOS (in 10% DMSO/10% Cremophor EL/80% PBS), or MBOSNP (in 100% PBS) via lateral tail vein at 8 mg/kg treatment concentration, and then euthanized by CO_2_ inhalation at selected time points and blood sample obtained by cardiac puncture. Plasma was processed from whole blood by centrifugation of EDTA-treated blood for 10 minutes at 9,600*g*, then stored at −80°C until analyzed. In addition, kidneys, spleen, lung, liver, and brain were removed, weighed; flash frozen, then homogenized in a 3-fold volume of PBS for further analysis and stored at −80°C. For standard curve construction, 100 µL of blank plasma (Bioreclamation, LLC) or tissue homogenate was infused with 2 µL of varying concentrations of MB or MBOS, then processed as described below. Next, 100 µL of each sample (plasma or tissue homogenate) was crashed with 200 µL methanol+0.1% formic acid+50 ng/mL (final concentration) n-benzyl benzamide internal standard, vortexed for 15 seconds, incubated at room temperature for 10 minutes, then centrifuged at 16,000*g* for 5 minutes at 4°C. 250 µL of supernatant was then transferred to an Eppendorf tube, centrifuged as previously described, then 195 µL of supernatant transferred to an HPLC vial with insert for analysis by HPLC-MS/MS (AB Sciex 3200 QTrap). An Agilent C18 XDB column (50 × 4.6 mm, 5 micron packing) was used for chromatography with the following conditions: 0–1.5 minutes in 90% Buffer A (water+0.1% formic acid), 1.5–2 minutes in 100% Buffer B (acetonitrile+0.1% formic acid), 2–3.5 minutes in 100% Buffer B, and 3.6–4.5 minutes in 90% Buffer A. MB was detected with the mass spectrometer in MRM (multiple reaction monitoring) mode by following the precursor to fragment ion transitions: 284.1/268.0 (intact MB) and 270.0/254.0 (demethylated MB), while the internal n-benzyl benzamide was detected using a 212.1/91.1 transition. The limit of detection (LOD) was set at the standard concentration providing an analyte signal three-fold above blank matrix. The limit of quantification (LOQ) was set at the lowest standard concentration which upon back-calculation gave a measured concentration within 20% of nominal and which was above the LOD. Per UTSW’s lab SOPs, samples falling below LOQ and above LOD are assigned a value of ½LOQ. Samples falling below LOQ and LOD are assigned a value of 0. Tissue concentrations of MB were corrected by subtracting residual compound remaining in the vasculature. Reference values for tissue vasculature provided in Kwon, 2001 were used for this calculation [56]. Pharmacokinetic parameters for MB were calculated using the noncompartmental analysis tool in Phoenix WinNonlin.

### Statistical analysis

All data are given as the means ± S.E. The difference in significance among groups with one independent variable was determined by one-way ANOVA with Dunnett’s multiple comparisons test for intended comparisons between groups when significance was identified. The difference in significance among groups where two independent variables occurred was established by two-way ANOVA with Dunnett’s multiple comparisons test for arranged comparisons between groups when significance was identified. For all tests, p < 0.05 was deemed significant.

## Results and Discussion

### Characterization of MBOSNPs

Following analysis of numerous batches of MBOSNPs, an average particle size of 166.95 ± 63.1 nm was determined. Based on data reported by other publications, the value of our nano-formulations was determined to be within an acceptable range compared to other MB- or MBOS-loaded nanoparticle formulations (Table 1) [57–59]. Additionally, the size distributions (PDI) associated with the formulations ranged from 0.287 to 0.387, allowing for consistent particle size within and among the batches. Due to the small size and relatively uniform distribution of the NP formulations, enhanced cellular uptake and rapid passage through the bloodstream to the target tissue is expected. Finally, the zeta potential of the drug-loaded nanoparticles exhibited values of approximately −32 mV (Table 1). Nanoparticles exhibiting surface charge values between −1 and −45 mV have been shown to increase the likelihood of BBB permeation, as well as enhance their stability [60]. These values were also determined for empty, blank nanoparticles and deemed comparable to the drug-loaded formulation (at −38.33 ± 11.66 mV) (Table 1). Simultaneously, the average drug loading and encapsulation efficiencies for MBOSNPs were also determined to be ~2% and 29% (Table 1), respectively. These values were also found to be comparable to previously published nanoformulations containing MB or MBOS [57–59].

**Figure 2:**
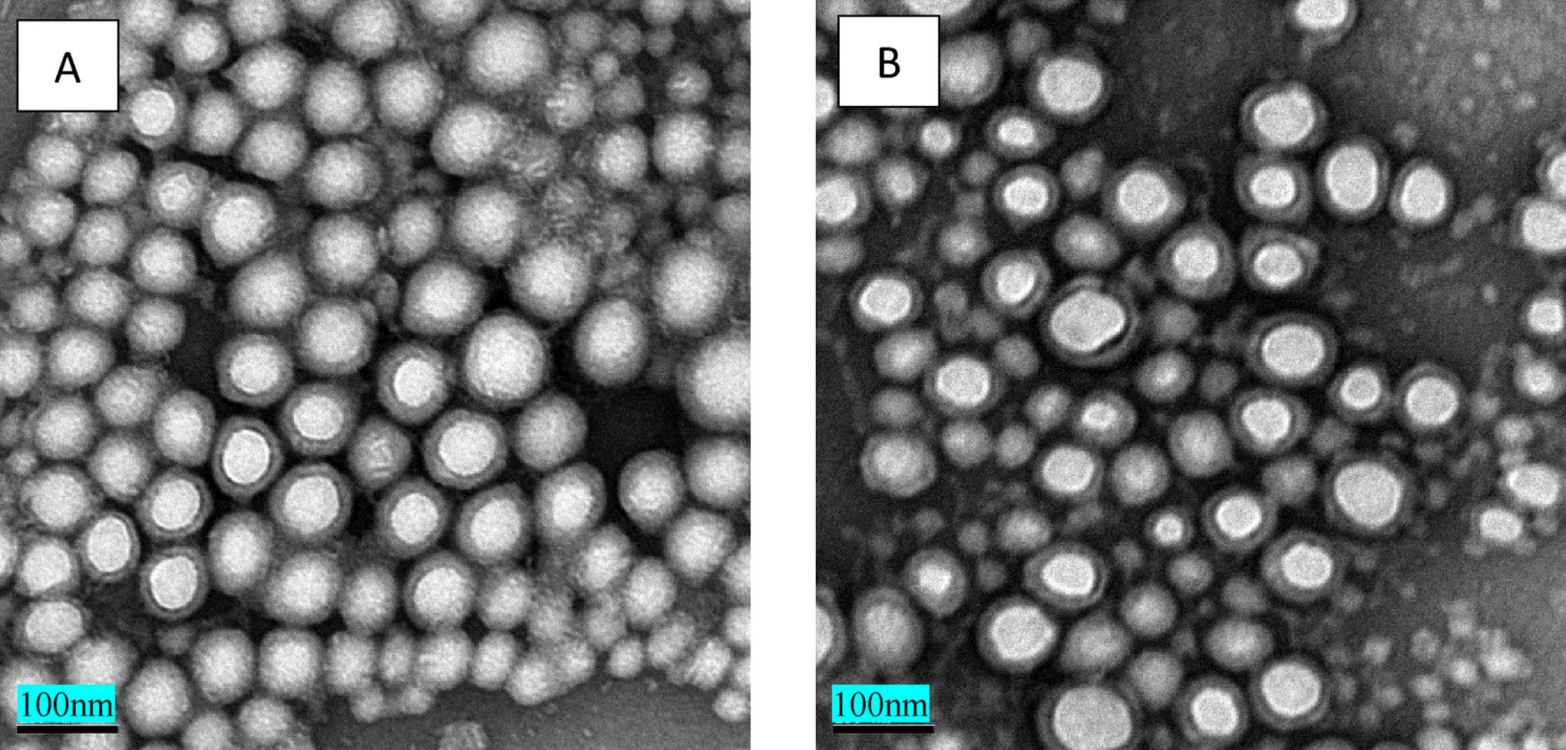
TEM images of Blank NPs (A) and MBOSNPs (B). Lyophilized NPs were resuspended in DDI H_2_O, then further diluted 1:10 in DDI H_2_O and processed by negative staining for analysis. Both sets of NPs display a spherical shape with a dark, outer ring depicting the Pluronic F68 layer and lighter, inner core of PLGA. Scale bar set to 100 nm. Abbreviations: MBOSNPs, methylene blue oleate salt-loaded polymeric nanoparticles; NPs, nanoparticles; DDI, distilled, deionized; PLGA, poly (D,L-lactide-co-glycolide).

**Figure 3:**
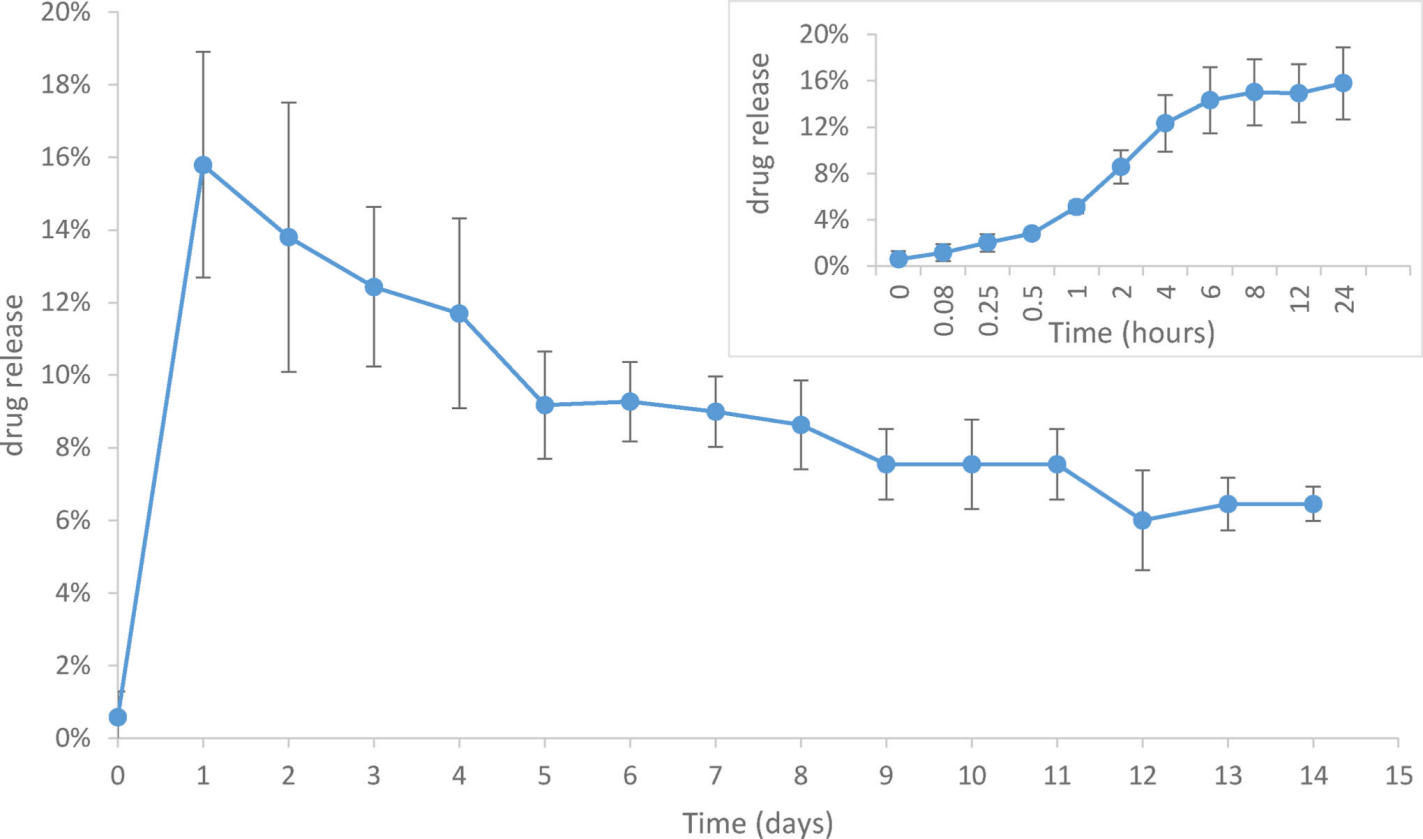
Cumulative Release Kinetic Profile of MBOS from MBOSNPs. Following addition of drug-loaded NPs to PBS in 8MWCO dialysis tubing, PBS from “sink” was collected at the designated time points at 28°C under constant stirring. Peak release occurred at 24 h (~16%) and gradually decreased until final sample collection at 14 days. Decrease in MBOS release possibly due to conversion of MBOS to MB, then LMB due to exposure to light, resulting in variability in quantified concentration by UV/Vis spectrophotometry. Inset graph illustrates %MBOS release values between 0 and 24 hours. N=3. Abbreviations: MBOS, methylene blue oleate salt; MBOSNPs, methylene blue oleate salt-loaded polymeric nanoparticles; NPs, nanoparticles; PBS, phosphate buffered saline; MB, methylene blue; LMB, leucomethylene blue.

In addition to the aforementioned physicochemical characterizations of MBOSNPs and BNPs, the size and surface morphology were evaluated by TEM, as illustrated in Figure 2, with a subsequent release kinetic profile obtained, as shown in Figure 3. From TEM analysis, we were able to obtain a graphical representation of the overall, and expected, spherical morphology of the blank and drug-loaded NPs, as well as confirm the size and size distribution data acquired from the previously performed physicochemical analysis. Following formulation and preliminary characterization of MBOSNPs, release kinetic studies were also performed in order to determine the peak release, as well as overall release profile. Several batches of drug-loaded NPs with similar physicochemical characteristics were analyzed to obtain uniform data. After analysis concluded at 14 days, the peak drug release was found to occur at 24 hours, with a gradual reduction in release over time (Figure 3).

### MBOSNPs impair cellular metabolism in U87 and T98G cells

**Figure 4:**
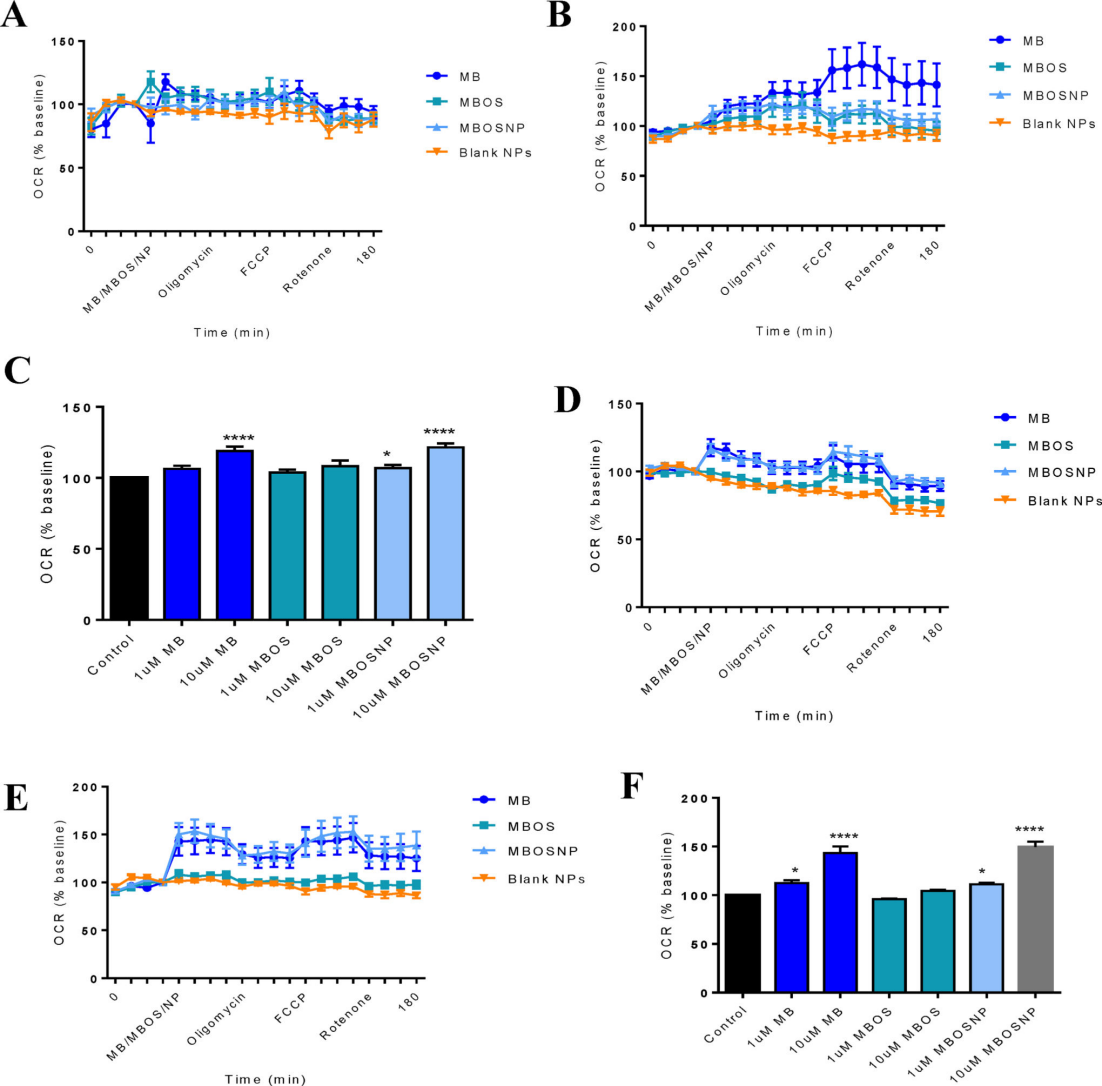
MBOSNPs increase OCR in U87 and T98G cells. A and B, OCR of U87 cells following 1 and 10 µM treatments, including BNPs, respectively. C, MB and MB (OS) NPs significantly increased U87 cell OCR at 10 µM. D and E, OCR of T98G cells following 1 and 10 µM treatments, including BNPs, respectively. F, MB and MBOSNPs significantly increased T98G cell OCR at 1 and 10 µM. N=3. *p<0.05 and ****p≤ 0.0001 by 1-way ANOVA and Dunnett’s multiple comparisons test. Abbreviations: MBOSNP, methylene blue oleate salt loaded polymeric nanoparticles; OCR, oxygen consumption rate; BNPs, blank polymeric nanoparticles.

We assessed the effects of MB, MBOS, and MBOSNPs on metabolic processes and their by-products at concentrations between 100 nM and 10 µM in U87 and T98G cells. In U87 cells treated with 10 µM MB or MBOSNP, there was a significant increase in oxygen consumption rates (OCR) (Figure 4B and 4C), which was not seen with the lower treatment concentration (Figure 4A and 4C). However, with T98G cells, the increase in OCR following 10 µM MB and MBOSNP treatments was more pronounced at 1.5-fold (Figure 4E and 4F), with an additional noted increase with both 1 µM treatments (Figure 4D and 4F).

**Figure 5:**
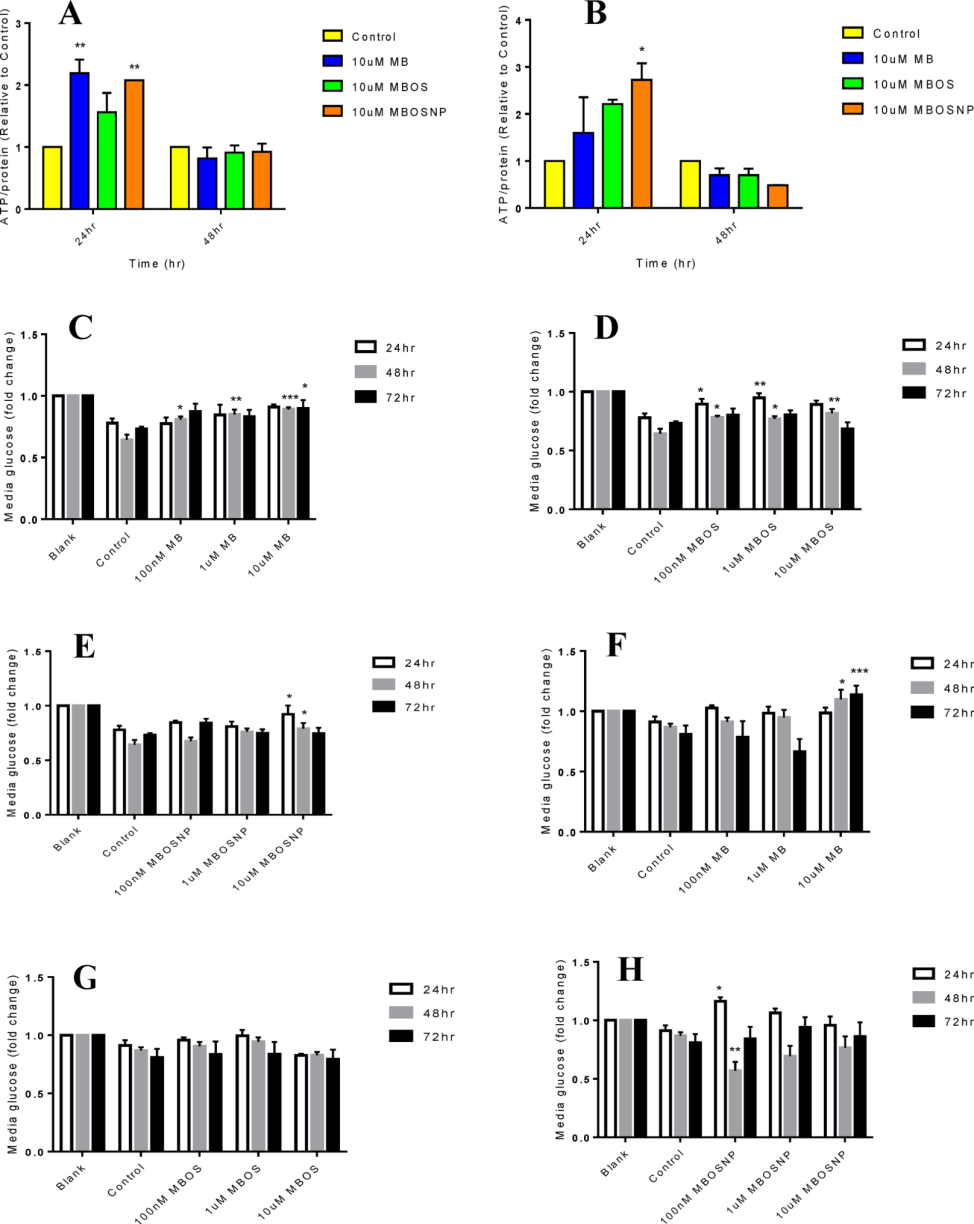
MBOSNPs alter cellular bioenergetics. A, MB and MBOSNPs increased ATP production at 24 h in U87 cells. B, MBOSNPs increased ATP production at 24 h in T98G cells. C–E, Effects of varying concentrations of MB, MBOS, and MBOSNPs on glucose quantification in U87 cells at 24, 48, and 72 h. F–H, Effects of varying concentrations of MB, MBOS, and MBOSNPs on glucose quantification in T98G cells at 24, 48, and 72 h. N=4. *p<0.05, **p ≤ 0.01, ***p ≤ 0.001 by 2-way ANOVA and Dunnett’s multiple comparisons test. Abbreviations: MBOSNPs, methylene blue oleate salt-loaded polymeric nanoparticles; MB, methylene blue; ATP, adenosine triphosphate; MBOS, methylene blue oleate salt.

To determine the long-term effects of MBOSNPs on cellular bioenergetics, we treated U87 and T98G cells with 10 µM MB, MBOS, and MBOSNPs for 24, 48, or 72 hours and then measured ATP production (24 or 48 hours) and glucose utilization (24, 48, or 72 hours). ATP levels were increased in 24 hours in both cell lines with 10 µM MBOSNP, and 10 µM MB in U87 cells compared to control (Figure 5A and 5B). However, an increase in ATP production was not observed by 48 hours, possibly due to a reduction in cell viability and/or number. When quantifying sample glucose based on the original media glucose concentration (4.5 g/L), all 10 µM treatments resulted in significant increases at 48 hours compared to untreated cells in U87 cells (Figure 5C through 5E), but only in 10 µM MB at 48 and 72 hours in T98G cells (Figure 5F through 5H). Cell fitness and number may also be implicated in the lack of significant glucose utilization after 48 hours in all 10 µM treatments, regardless of cell line.

### MBOSNPs inhibit *in vitro* tumor growth

**Figure 6:**
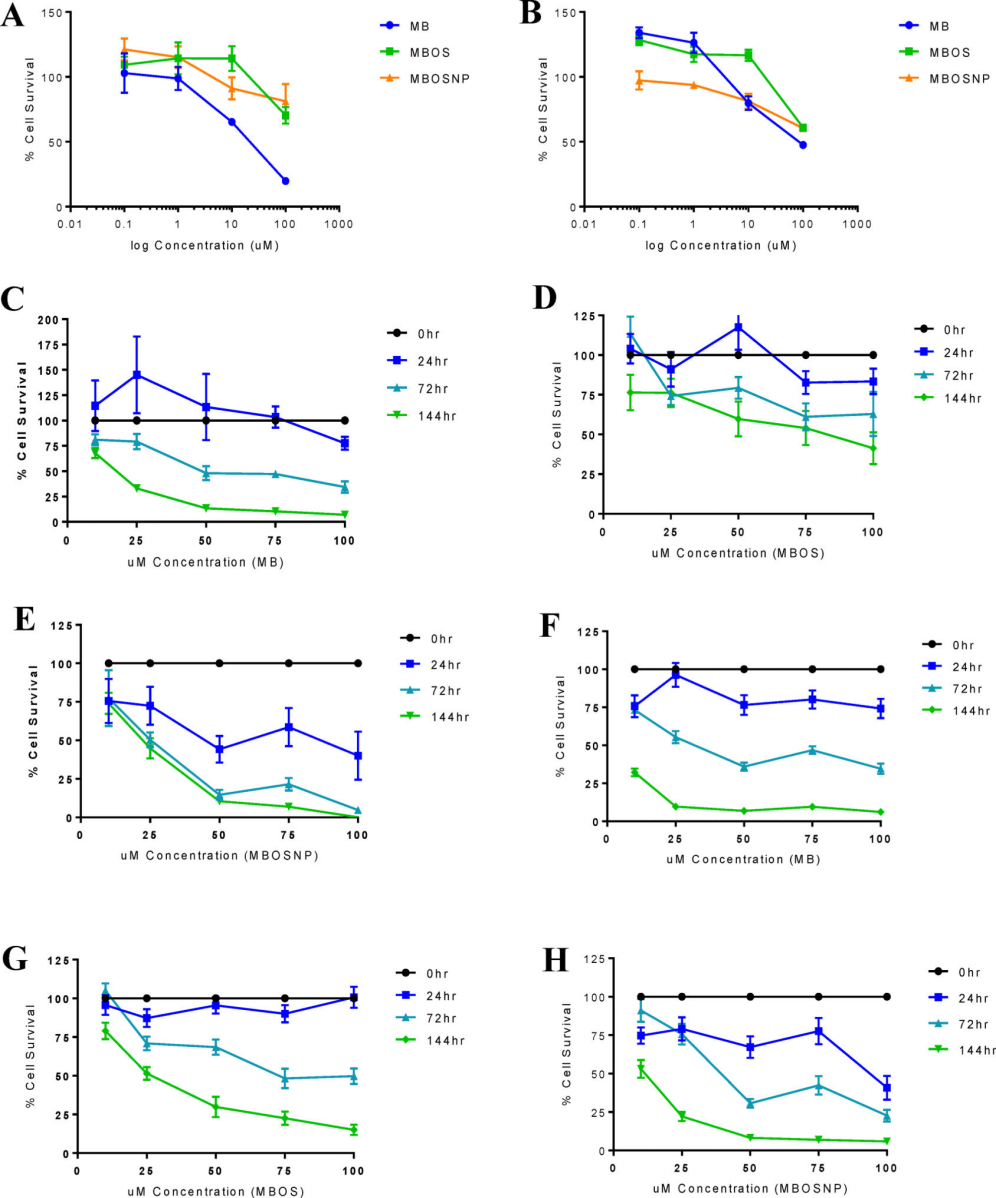
MBOSNPs inhibit U87 and T98G cell survival. A and B, Cell survival analysis at 96 h post treatment in U87 and T98G cells, respectively. C–E, U87 cell survival following varying MB/MBOS/MBOSNP treatment concentrations at 24, 72, and 144 h. C, Reduction in viability not noted at lowest concentration (10 µM) until 72 h. D, U87 cell viability reduced at lowest concentration at 144 h. E, U87 cell viability reduced at lowest concentration beginning at 24 h. F–H, T98G cell survival following varying MB/MBOS/MBOSNP treatment concentrations at 24, 72, and 144 h. F, T98G viability reduced at lowest concentration beginning at 24 h, with drastic reduction at 144 h. G, T98G viability not noted until 144 h H, T98G cell viability reduced following lowest treatment concentration starting at 24 h, with drastic reduction at 144 h. Based on these data, the IC50 for MBOSNP was 10 µM. Additionally, MBOSNP treatment seemed to demonstrate a comparable, if not more effective, reduction in cell viability to free MB. N=6. Abbreviations: MBOSNPs, methylene blue oleate salt-loaded polymeric nanoparticles; MB, methylene blue; MBOS, methylene blue oleate salt.

To elucidate how changes in GBM metabolism and bioenergetics following MBOSNP treatment influenced tumor growth, we assessed cell viability and anchorage-dependent colony formation in U87 and T98G cells. Based on preliminary studies (unpublished), neither cell line exhibited a significant reduction in cell viability until 96 hours when analyzing a concentration range from 100 nM to 100 µM of MB, MBOS, and MBOSNPs (Figure 6A and 6B). Once the IC_50_ dose range was narrowed down to between 10 and 100 µM, an extended time course was performed. In T98G cells treated with 10 µM MB or MBOSNPs for 24 and 72 hours, there was an initial reduction in cell viability by 25% that stayed consistent through 144 hours with MB(OS)NP treatment, but increased by an additional ~50% following MB treatment (Figure 6F and 6H). However, with the same treatment concentration for MBOS, there was no significant reduction in T98G cell viability until 144 hours (Figure 6G). With U87 cells, 10 µM MB did not induce cell death until 72 and 144 hours, with an increase in cell viability following a 24-hour treatment (Figure 6C). According to previously published data, low concentrations of MB have been shown to enhance cell viability by augmenting mitochondrial function [31], which could explain this observation. A similar trend in cell viability following MB treatment was also noted with MBOS treatments, with a reduction in cell survival not seen until 144 hours (Figure 6D). However, in U87 cells treated with even the lowest concentration of MBOSNPs (10 µM), a significant reduction in cell viability was detected at all three time points (Figure 6E). In both cell lines, the IC_50_ for MBOSNP treatment was determined to be 10 µM, which was observed to induce similar, if not better, reduction in cell viability when compared to free MB, but especially free MBOS.

**Figure 7:**
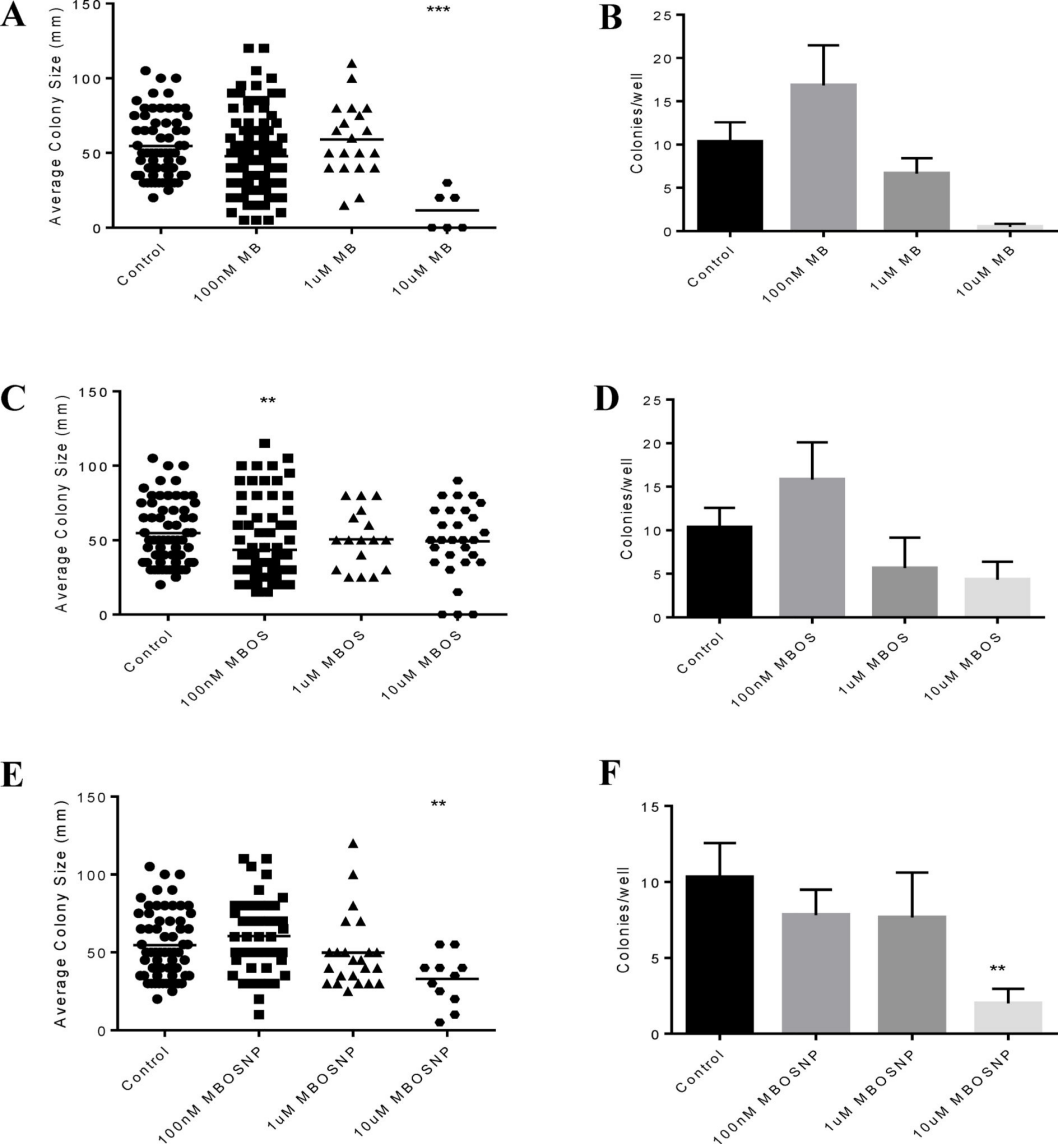
MBOSNP treatment inhibits U87 cell proliferation. A, 10 µM MB treatment significantly reduced average colony size compared to control. B, 10 µM MB treatment reduced average number of U87 colonies. C, 100 nM, but not 1 or 10 µM MBOS, treatment significantly reduced average colony size compared to control. D, MB treatment all concentrations had no effect on average number of U87 colonies. E, 10 µM MBOSNP treatment significantly reduced average colony size compared to control. F, 10 µM MBOSNP treatment reduced average number of U87 colonies. N=3. **p ≤ 0.01, ***p ≤ 0.001 by 1-way ANOVA and Dunnett’s multiple comparisons test. Abbreviations: MBOSNP, methylene blue oleate salt-loaded polymeric nanoparticles; MB, methylene blue; MBOS, methylene blue oleate salt.

**Figure 8:**
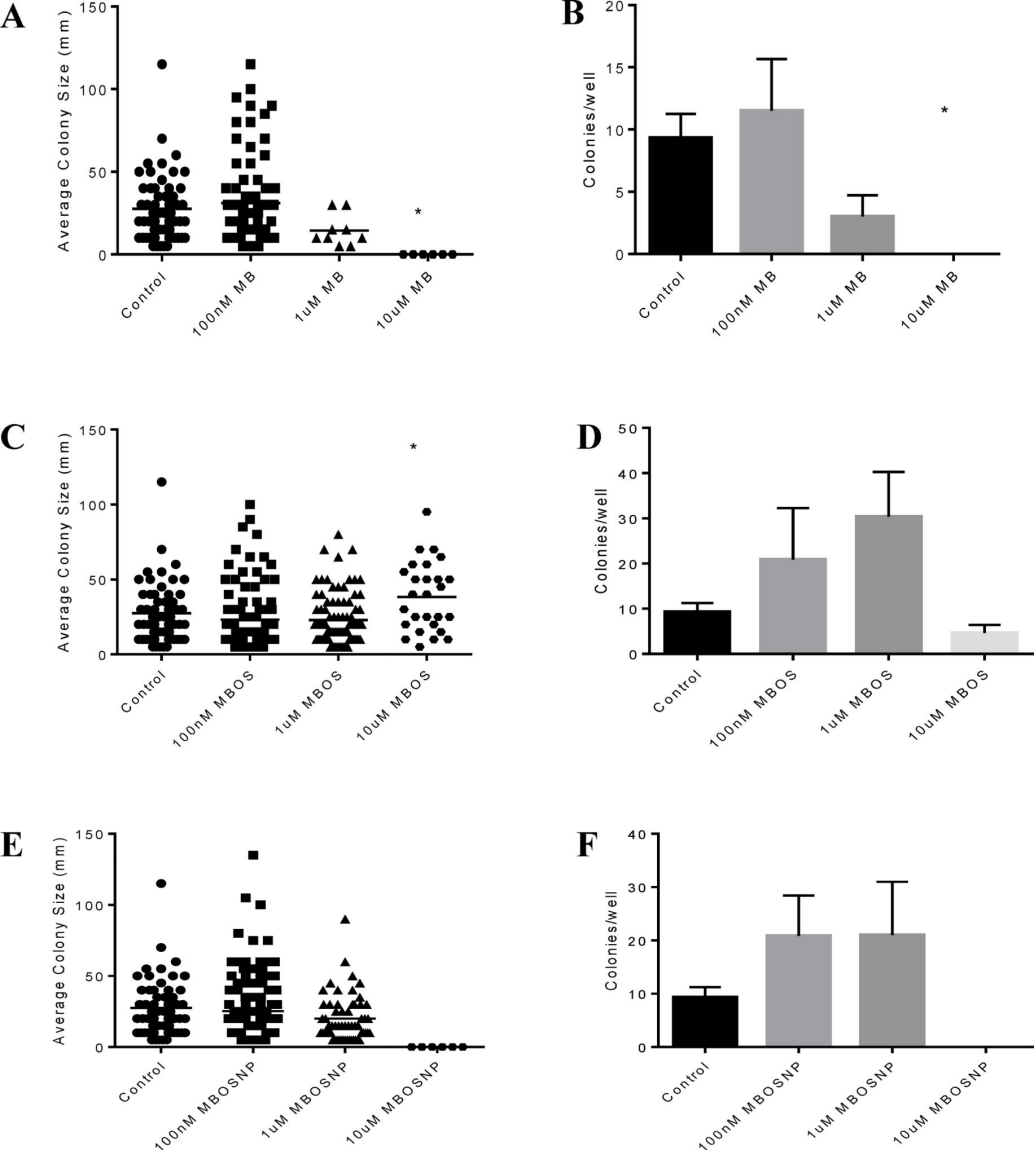
MBOSNP treatment inhibits T98G cell proliferation. A, 10 µM MB treatment significantly reduced average colony size compared to control. B, 10 µM MB treatments significantly reduced average number of T98G colonies. C, 10 µM MBOS treatment significantly increased average colony size compared to control. D, MB treatment all concentrations had no effect on average number of T98G colonies. E, 10 µM MBOSNP treatment significantly reduced average colony size compared to control. F, 10 µM MBOSNP treatments reduced average number of T98G colonies. N=3. *p<0.05, **p ≤ 0.01 by 1-way ANOVA and Dunnett’s multiple comparisons test. Abbreviations: MBOSNP, methylene blue oleate salt-loaded polymeric nanoparticles; MB, methylene blue; MBOS, methylene blue oleate salt.

In addition to cell viability analysis, U87 and T98G cells were treated as previously described to determine the effects of MBOSNPs on cell proliferation. Following a 4-week liquid colony formation assay and subsequent examination of the average size and number of colonies, MBOSNP treatment was shown to be as effective as free MB, and more effective than free MBOS, at inhibiting cell proliferation in U87 and T98G cells at identical treatment concentrations (Figures 7 and 8, respectively). In both cell lines, the colonies obtained following 10 µM MB and MBOSNP treatments were on average fewer and smaller than their untreated, control cell counterparts (Figures 7 and 8). For U87 cells, both colony size and number were significantly reduced following 10 µM MBOSNP treatment (Figure 7E and 7F), while only size was reduced to a comparable level by the same MB concentration (Figure 7A). In U87 cells treated with MBOS, little to no difference in colony size and number of colonies was produced regardless of treatment concentration compared to untreated U87 cells (Figure 7C and 7D). In T98G cells, all treatments produced similar outcomes as occurred with U87 cells, such that 10 µM MB significantly reduced the average size and number of T98G colonies (Figure 8A and 8B), while the same concentration of MBOSNP only significantly impacting average size, but not colony number (Figure 8E and 8F). However, 10 µM MBOS did induce a change in average T98G colony size, but in the inverse direction (Figure 8C), which was also seen but with the lowest concentration in U87 cells. Supplemental Figures 1 and 2 further illustrate the difference in average colony size and number of colonies for each treatment and concentration. Based on these statistical and graphical data, MBOSNP treatment demonstrated an ability to not only inhibit GBM cell viability, but also proliferation.

### Bio-distribution of MBOSNPs

We further determined the bio-distribution of our nanoformulations. Following an initial analysis of MB and MBOS by LC-MS/MS, we found that both compounds produced chemical peaks at 284 and 270, but with inverted component ratios (i.e. MB had a 20:1 ratio of 284:270 mw species, while MBOS had a 15:1 ratio of 270:284 mw species). Through further evaluation, it was determined that the 284 mw species was intact MB, while the 270 mw species was demethylated at some position on MB. While the difference in species ratios in MB and MBOS did not appear to result in functional inactivity *in vitro*, a similar assumption was reached in regards to *in vivo* effects.

**Figure 9:**
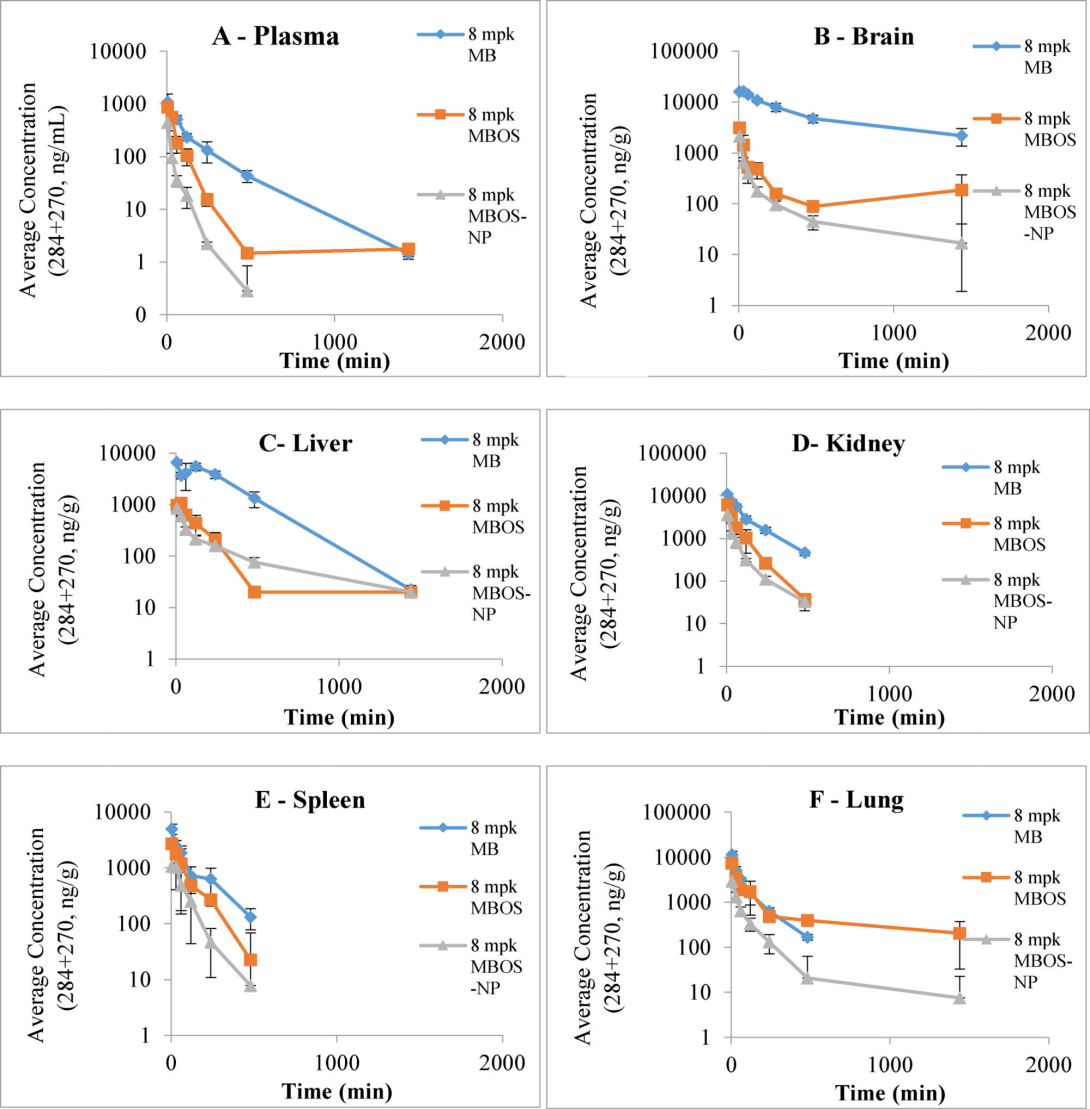
Bio-distribution of MB, MBOS, and MBOSNPs at 8 mg/kg (8 mpk) drug concentration. Following administration of each treatment, blood and tissues were collected from CD-1 mice and analyzed by LC-MS/MS for 284 and 270 mw species (intact versus demethylated MB) at designated time points. The ng/mL or g drug concentrations per plasma volume or tissue weight, respectively, was obtained and plotted according to WinNonlin Noncompartmental PK parameters. In all samples, free MB had the highest concentration, followed by free MBOS, then MBOSNPs. Abbreviations: MB, methylene blue; MBOS, methylene blue oleate salt; MBOSNPs, methylene blue-oleate salt loaded polymeric nanoparticles; mpk, mg/kg; LC-MS/MS, liquid chromatography-mass spectrometry/mass spectrometry (liquid chromatography tandem mass spectrometry); mw, molecular weight; PK, pharmacokinetics.

Following treatment administration and tissue collection, free MB exhibited the highest drug concentrations across the designated time points, regardless of tissue type (Figure 9 and Table 2). However, due to the aforementioned issue involving the differences in mw species ratios for MB and MBOS, it was determined that the best comparison would be between concentrations of free MBOS and MBOSNPs recovered from the plasma and tissues. Upon doing so, it was determined that overall, MBOS exhibited better tissue accumulation following administration compared to MBOSNPs containing the same MBOS concentration (Figure 9 and Table 2). However, using the drug concentration values obtained (area under the curve – AUC), it was established that MBOSNPs were more effective at crossing the BBB than free MBOS by 1.6-fold (Table 2), due to the presence of the PF68 coating. As a result, this provides for a potential application for the drug-loaded NPs as a GBM therapy upon further formulation optimization and scale up.

## Conclusions

Due to the aggressive nature of GBM that results in sub-standard survival rates beyond two years, development of new therapies is required in order to more effectively treat the disease. While a cure for GBM is unlikely in the near future, advancements toward better identifying genes responsible for its growth and progression are possible. As a result, the utilization of formerly rejected compounds could be beneficial in treating GBM, if proper delivery systems are employed.

In this study, we were able to repurpose the photosensitizer methylene blue as a potential GBM chemo-therapeutic, by encapsulating it within nanoparticles composed of the synthetic co-polymer, PLGA. These NPs, which were formulated through solvent displacement, were coated with PF68 to enhance BBB passage, and then analyzed by DLS for particle size, size distribution (PDI), and zeta potential (surface charge). Additionally, particle size and surface morphology were analyzed by TEM, with drug loading and encapsulation efficiency performed by UV-Vis at 645 nm. The resultant drug-loaded PLGA NPs obtained were below size restrictions for passage across the BBB (<200 nm), which was confirmed by their enhanced ability to permeate the BBB compared to free MBOS and accumulate in the brain tissue, even at lower concentrations than free MBOS. Furthermore, MBOSNPs induced comparable, if not better, levels of cell death and inhibition of cell proliferation to free MB and MBOS, while inducing similar or greater degrees of cellular and metabolic changes in U87 and T98G cells *in vitro*, demonstrating their potential application as a treatment for GBM.

However, due to the variability in mw species ratios between MB and MBOS/MBOSNPs, a definitive comparison of their accumulations was not possible. Thus, this precluded an accurate determination of the effectiveness of MBOSNPs compared to free drug in vivo. With that said, we were able to determine that MBOSNPs were more efficient at permeating the BBB than free MBOS, establishing their potential application as a neuro-therapeutic for GBM. However, toxicological studies on free MBOS (and MBOSNPs) are imperative, as there is currently no data available on how the compound might affect animals, and ultimately humans, upon administration. Nevertheless, a forthcoming xenograft mouse model study is necessary to fully elucidate if the outcomes demonstrated in cultured cells could be translated into a tumor-bearing animal.

## Supplementary Material

Suppl file

## Figures and Tables

**Table 1:** Physico-chemical characteristics of methylene blue oleate salt-loaded polymeric nanoparticles (MBOSNPs) compared to blank, non-drug-loaded polymeric nanoparticles (BNPs).

Nanoparticle Type	Average Particle Size	Average Polydispersity Index (PDI)	Average Zeta Potential	Average Drug Loading	Average Encapsulation Efficiency
**MBOSNPs**	166.95 ± 63.1 nm	0.287 ± 0.1	−32.12 ± 4.98 mV	2.21 ± 0.74%	29.16 ± 7.47%
**BNPs**	132.25 ± 17.99 nm	0.186 ± 0.06	−38.33 ± 11.66 mV	N/A	N/A

**Table 2:** PK study values for area under the curve (AUC) in min.ng/mL for plasma or min.ng/g for tissues.

Tissue	MB	MBOS	MBOSNP
Plasma	127,828 ± 7,507	52,659 ± 3,247	14,358 ± 1,109
Brain	7,626,056 ± 340,435	333,423 ± 46,799	142,258 ± 8,764
Liver	2,391,840 ± 162,972	172,265 ± 12,683	149,236 ± 5,744
Kidney	1,230,440 ± 40,621	423,681 ± 36,095	189,473 ± 6,653
Spleen	500,163 ± 41,386	244,660 ± 25,905	101,203 ± 15,207
Lung	765,373 ± 39,324	939,911 ± 76,188	187,453 ± 16,075
