# Supplementary material for: Development and Characterization of Methylene Blue Oleate Salt-Loaded Polymeric Nanoparticles and their Potential Application as a Treatment for Glioblastoma
Source: J Nanomed Nanotechnol. Author manuscript; Available in PMC 2017 Oct 11. (PMC5636194; doi:10.4172/2157-7439.1000449)
Supplement: Suppl file [file NIHMS907690-supplement-Suppl_file.pdf]

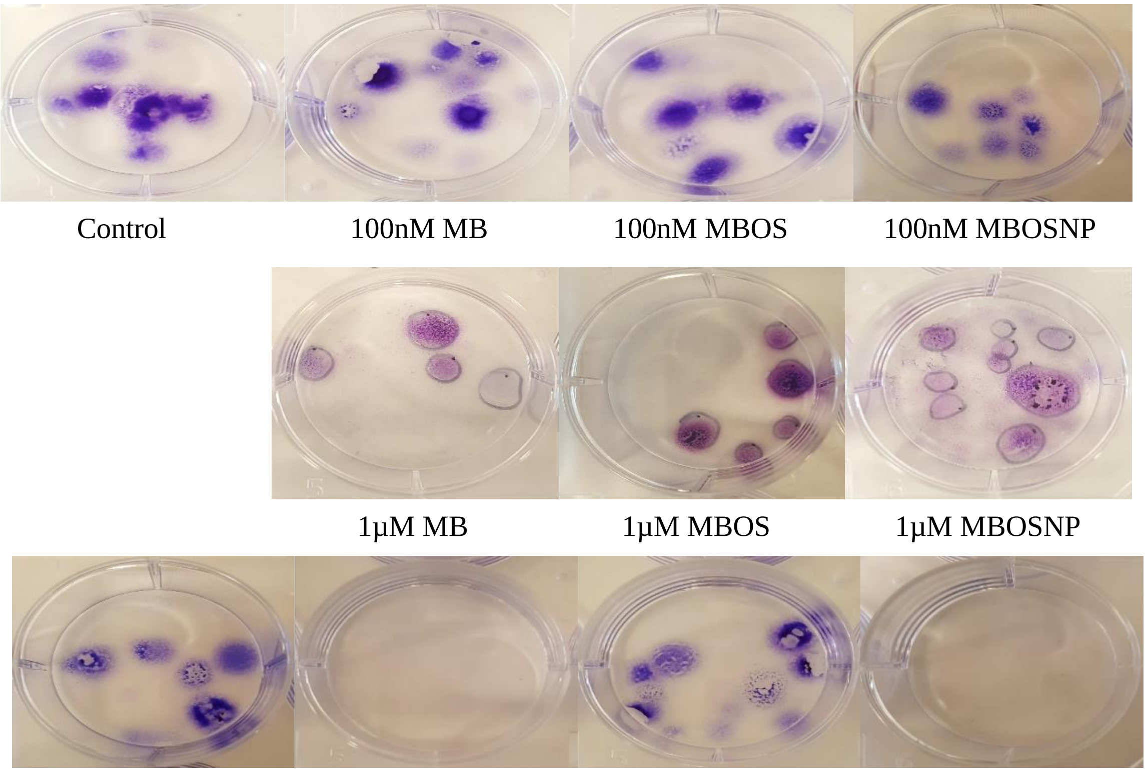

Supplemental Figure 1 – Effects of MBOSNPs on U87 cell proliferation. As treatment concentrations increased, average colony size and number of colonies decreased. At 10 µM MB and MBOSNP, little to no cells were detectable by visual examination, and those that were present were significantly smaller than untreated U87 cells. All MBOS treatment concentrations had similar sized colonies, as well as number of colonies. MBOSNP treatments compared to BNP-treated U87 cells to establish effect of PLGA, with no visible effect identified. Abbreviations: MBOSNPs, methylene blue oleate salt-loaded polymeric nanoparticles; MB, methylene blue; MBOS, methylene blue oleate salt; BNP, blank polymeric nanoparticles; PLGA, poly(D,L-lactide-co-glycolide).

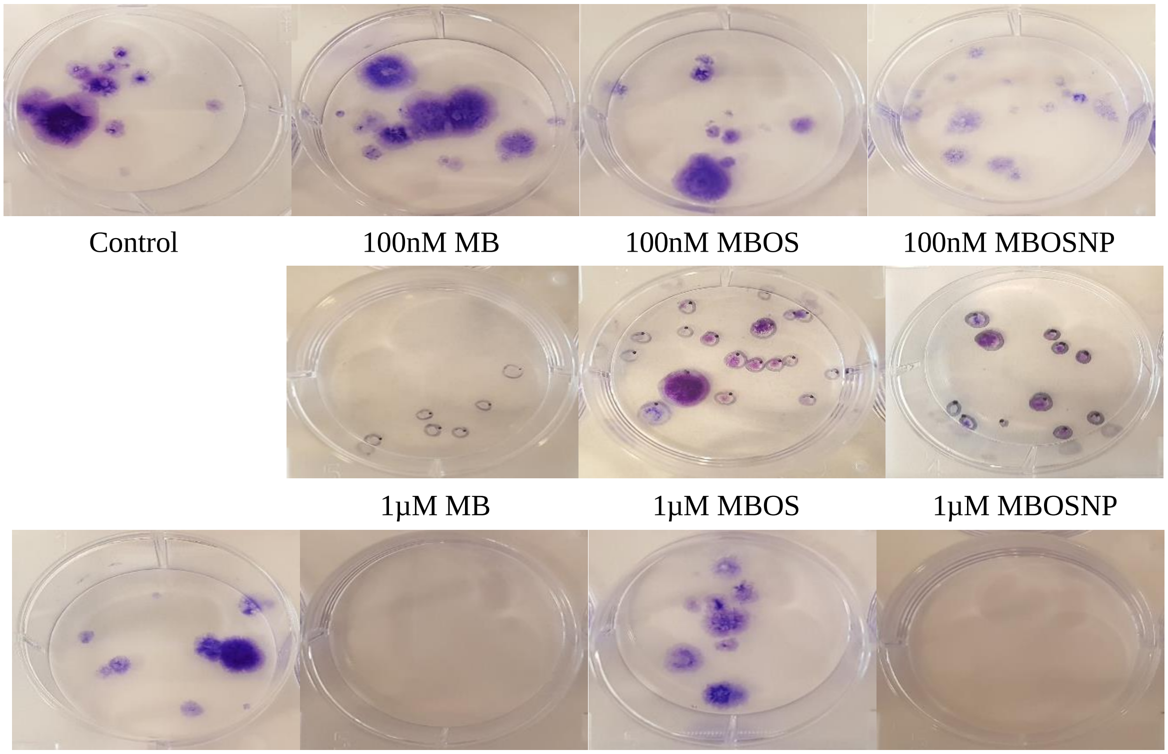

Supplemental Figure 2 – Effects of MBOSNPs on T98G cell proliferation. As treatment concentrations increased, average colony size and number of colonies decreased. At 10 µM MB and MBOSNP, little to no cells were detectable by visual examination, and those that were present were significantly smaller than untreated T98G cells. All MBOS treatment concentrations had similar sized colonies, as well as number of colonies. MBOSNP treatments compared to BNP-treated T98G cells to establish effect of PLGA, with no visible effect identified. Abbreviations: MBOSNPs, methylene blue oleate salt-loaded polymeric nanoparticles, MB, methylene blue, MBOS, methylene blue oleate salt; BNP, blank polymeric nanoparticles; PLGA, poly(D,L-lactide-co-glycolide).
